# A novel temporal classification prototype network for few-shot bearing fault detection

**DOI:** 10.1038/s41598-025-98963-4

**Published:** 2025-04-24

**Authors:** Yanfei Liu, Ziang Du, Hao Zheng, Qian Zhang, Cheng Chen, Nana Wu

**Affiliations:** 1 Department of Basic Courses, Xi’an Research Institute of Hi-Tech, 710025 Xi’an, China; 2 Experimental Training Base, National University of Defense Technology, 710106 Xi’an, China

**Keywords:** Mechanical engineering, Mathematics and computing

## Abstract

In the process of industrial production, bearing fault detection has always been a hot issudza20000528@163.comsolved. At present, the problem of less fault data samples in the field of fault detection has caused great trouble to the research of deep learning. In the application of industrial fault detection, which is difficult to obtain massive data, it is easy to lead to the lack of fitting of neural network training and many generalization problems. To solve the above problems, this paper proposes an improved and more efficient method of few-shot supervised learning, which is called the Temporal Classification Prototype Network (TCPN). This model is designed to maintain both training efficacy and generalization capabilities under conditions of data scarcity. Initially, Fourier transform is employed to accentuate the frequency domain characteristics of the fault section in the bearing signal before it is input into the model, thereby enabling the subsequent model to concentrate on distinguishing between normal and fault signals. Subsequently, discrete data sample points are transformed into points within the feature space via our Enhanced Temporal Convolutional Network(ETCN). In our investigation, we utilize the features of the support set as anchors within the feature space and employ similarity measures as the basis for classification, thus developing a more effective comparative learning classifier known as the ContractSim Classifier (CSC). Within the CSC, the model learns the data features of the query set, which are then back-propagated to refine our model. The proposed TCPN model has been evaluated across four standard bearing datasets, corroborating its few-shot learning proficiency through k-shot experiments. In comparative model experiments, our TCPN outperforms baseline models, while the ablation study confirms the rationality and robustness of our module integration.

## Introduction

Bearings are essential components in rotating machinery, widely used in various industrial equipment such as wind turbines, automobiles, airplanes, and production lines^1–5^. Due to its continuous operation in high-speed and heavy-duty environments, bearings are prone to failure, leading to decreased equipment performance or even shutdown^6^. According to industry statistics, about 40$\%$of failures in rotating machinery are caused by bearing failures^7,8^. Therefore, timely detection of bearing faults is of great significance for ensuring the normal operation of equipment and production safety.

The traditional bearing fault diagnosis is usually carried out under the professional knowledge of physical inspection, instrument monitoring and mechanical engineers^9^. In the era of big data and artificial intelligence, intelligent fault diagnosis undoubtedly opens up a new path for the field of fault diagnosis. As the main way of intelligent fault diagnosis, deep learning, especially Convolutional Neural Networks (CNN) and Recurrent Neural Networks (RNN), has achieved significant results in bearing fault detection due to its powerful feature extraction and pattern recognition capabilities^10–14^. However, deep learning models usually need a large number of data to train, and the lack of samples will lead to the problem of under fitting in the training process^15^. In addition, most of the current researches on deep learning have the problem of large demand for data resources. Few-shot learning is more suitable for the situation of data scarcity. In order to promote the deep combination of few-shot learning model and fault detection, some researches try to improve the generalization ability and robustness of the model by using transfer learning, data enhancement, meta learning and so on. Transfer learning improves the performance of new models by utilizing prior knowledge of existing models, but performance is highly dependent on the similarity of fault sample distributions^16,17^. Data augmentation expands training data by generating new samples, but model training is difficult and the problem of vanishing gradients is difficult to solve^18^.

As a few-shot learning technology, meta learning aims to improve the generalization ability and adaptability of the model by learning how to learn. In recent years, with the rapid development of deep learning model structure, meta learning has also made significant progress in the field of fault detection, and has shown great potential^19^. Meta learning enables the model to learn how to learn. This method in line with human learning style has been widely used in few-shot studies in recent years^20^. Some researchers began to use meta learning method to solve the problem of fault detection and deep learning fusion^21^.

In the recent research on bearing fault detection with meta learning, the importance of Fourier transform for feature representation is often ignored^22,23^. Moreover, with a small amount of data learned from few-shots, the final classification effect has higher requirements on the feature extraction ability of the model. Therefore, the quality of the feature extractor largely determines the learning ability of the model. Under the training of normal scale samples, TCN network has outstanding feature extraction ability in time series data^24,25^, including the subsequent improved TCN model, which has shown better effect than the conventional model in feature extraction task of fault detection^26–29^. However, many researches on few-shot learning ignore its ability of feature extraction in meta learning. In fact, its original feature extraction ability can continue to play in meta learning only by making some innovative design of its model framework. Our research is devoted to this work.

**Fig. 1:**
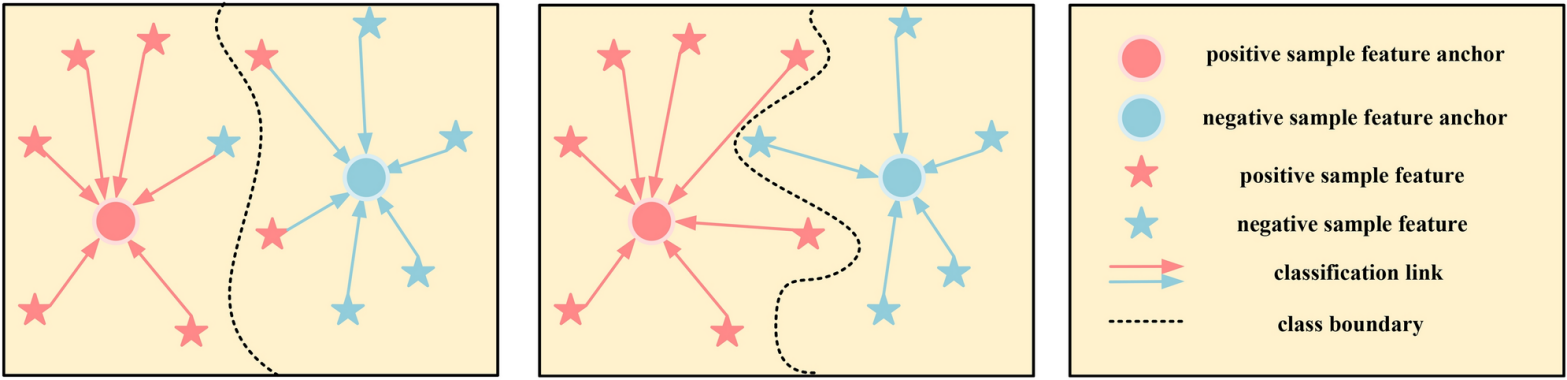
Distance based classification is often unable to classify correctly due to small deviations (Left). Classification based on similarity can solve this problem from the essence (Middle).

For the construction of classifiers, metric learning has the ability to learn low dimensional feature space, but it needs to select appropriate measurement function^30^. Moreover, metric learning can enhance the robustness and generalization of deep learning model through the distance of feature space^31^. As a network model in metric learning, prototype network can undoubtedly improve its classification ability by designing better classifiers, but its classifier is still limited to the conventional distance measurement in recent years, such as Euclidean distance, Manhattan distance, Chebyshev distance and so on^32–34^. However, The scarcity of samples will lead to the position error of feature anchor points, resulting in the wrong classification as shown in Fig. 1.

In our research, we design the framework of this model training based on the training idea of prototype network in meta learning. The whole model combines the characteristics of Fourier transform highlighting fault frequency domain features with the excellent convolution of time series signals in TCN to extract features. In addition, our prototype network breaks through the conventional distance classifier and designs the classifier according to the similarity between data samples and anchor points. Figure 1 is the schematic diagram of our classification method, which clearly shows the classification problems that need to be solved urgently in many current researches. Our research aims at more efficient feature extractors, more effective classifiers and more suitable frameworks for sample learning.

This research combines three modules suitable for improving the effect of fault detection, and designs our model TCPN in this paper. The contributions of our research can be briefly summarized as follows:

- In order to solve the problem that the bearing fault signal is difficult to be effectively processed due to the scarcity of data in the real situation, we propose to highlight the frequency domain characteristics of the fault signal by using Fourier transform to improve the learning effect of meta learning.
- Based on the fact that the existing research failed to design the feature extractor from the characteristics of the signal itself, we designed our feature extractor, Enhanced Temporal Convolutive Network (ETCN) based on the theory of Temporal Convolutive Network. While retaining the advantages of causal convolution, it also has the advantages over conventional time-series networks such as RNN and LSTM, and can capture the detailed information and global structure in the sequence data.
- Starting from how to improve the classification effect, this study designed a novel contract learning classifier, called ContractSim Classifier(CSC). It discards the original distance measurement and uses cosine similarity to measure the similarity between anchor points and sample feature points. We reasonably design the calculation method, and integrate the cosine similarity into the loss function and training process more efficiently, which makes the meta learning classification under the condition of data scarcity have updated progress.

The structure of the remainder of this article is as follows: Section 2 reviews the related work preceding this study. Section 3 delineates the key principles of our network and the training strategy for few-shot learning in detail. Section 4 involves an analysis and comparison of several datasets and prominent deep learning models, affirming the generalizability and effectiveness of our theoretical contributions. Finally, Section 5 concludes the paper with a summary of our findings.

## Related work

### Fault detection

Bearing fault detection technology can help operators and maintenance personnel find potential faults in advance, so as to take corresponding maintenance measures to avoid high maintenance costs and production losses caused by sudden faults^35^. Traditional bearing fault detection methods mainly rely on human experience to identify abnormal sounds and vibrations through hearing and touch^36^. However, this method has strong subjectivity, low accuracy, and can not adapt to the increasingly complex industrial environment. With the development of signal processing technology and machine learning algorithm, especially deep learning algorithm, bearing fault detection method based on vibration signal has gradually become a research hotspot^37^. At present, the cross integration of computer science and mechanical control is increasingly close. The method of in-depth learning can use sensors to collect the vibration data of bearing operation, and identify the fault characteristics through advanced data analysis technology, so as to achieve better results^38^. The Two-Stage Universal Domain Adaptation approach proficiently delineates multiple unlabeled novel fault types from the labeled familiar fault categories, while also enabling the determination of the quantity of new fault types present^39^. SDCGAN model overcomes the traditional model of multi-source domain data, and can effectively deal with the problems in new fields^40^.

In the past, the theory of fault signal has been fully studied, but the traditional fault detection technology is obviously not as efficient as intelligent fault detection in the era of intelligence. Intelligent fault detection is more suitable for data-driven intelligent diagnosis instead of manual detection. This not only greatly improves the efficiency and accuracy of detection, but also helps to realize the automation mode in industrial production and manufacturing.

### Few-shot learning

With the combination of few-shot learning and fault detection, more and more researches have realized that few-shot learning is an intelligent method in very industrial fault scenarios. At present, few-shot learning has several promising research fields.They are data enhancement technology^41^, measurement based method^42^, transfer learning^43^and meta learning^44^.Data enhancement technology aims to improve the effect of the model at the data level, and expand the data or even the characteristics of the data through a small amount of data. To achieve a kind of learning with less data to reach the original level, or even exceed the original level. The more common method based on measurement is to use distance measurement or cosine similarity to make classifiers^32–34^. For example, in the prototype network, distance is often used to classify unknown feature points^32^. The transfer learning is to apply the model knowledge learned from the source domain dataset to the field of fault detection, which is very suitable for the case of small data set of fault detection^43,45^. Meta learning can quickly adapt to new fault types, because it has learned how to use a small number of samples for learning through training on a variety of tasks^46,47^. At present, the most popular method is the hybrid method of the above methods.Such as few-shot fault detection model based on multi feature fusion^48^, a cross machine small fault detection method based on asymmetric distribution measure^49^, and a few-shot fault detection method combining transformer and variational attention^50^, etc.

In the current situation of scarcity of fault signals in actual industrial production, few-shot learning has been deeply studied because of its unique advantages. A variety of few-shot learning methods are constantly adapted to the task of fault detection. Meta learning is one of the mainstream methods because it can make the deep learning model adapt to new fault types. In recent years, most of the research is a mixture of past research methods, aiming to make up for the defects of few-shot learning method through modular improvement.

### Prototype networks

In the past, the anchor point in the feature space was often used as the feature invariant point of the whole data sample in the Prototype Network(PN). The distance between many data and anchor points is used as the classification basis^32^. Many studies have confirmed that improving the feature extractor of the prototype network can improve the performance of fault detection. Xie et al^51^. improved two-dimensional convolutional neural network (2 d-cnn) as an embedded network, and then used PN to identify welding defects in materials. Tang et al^52^. introduced coordinate attention mechanism on the basis of continuous wavelet transform to generate three channel spectrum diagram, so as to improve PN performance. However, these improvements to the existing advanced network ignore the characteristic that the bearing signal data is time series data. Li et al^53^. proposed a Reweighted Regularized Prototypical Network(RRPN) to solve the difficulty of learning discriminant metric space. By using the intra class and inter class information, similar samples are closely mapped together, while dissimilar samples become distant, so the query data outside the sample can be better classified. In this way, RRPN has hope in dealing with the FSFD problem of data scarcity. For the first time, it breaks the limitations of the past prototype network for distance classifier, but it does not perform well in the final experimental effect.

As the earliest network in meta learning, prototype network has unique advantages in dealing with fault diagnosis in the case of data scarcity. However, the design of the classifier of the prototype network is often limited to the distance, and most studies only make deep improvements in the feature extractor, ignoring the importance of the classifier. Even though a few studies have made breakthroughs, they have not achieved a good model effect because of the irrational design.

## Methods

### Framework overview

**Fig. 2:**
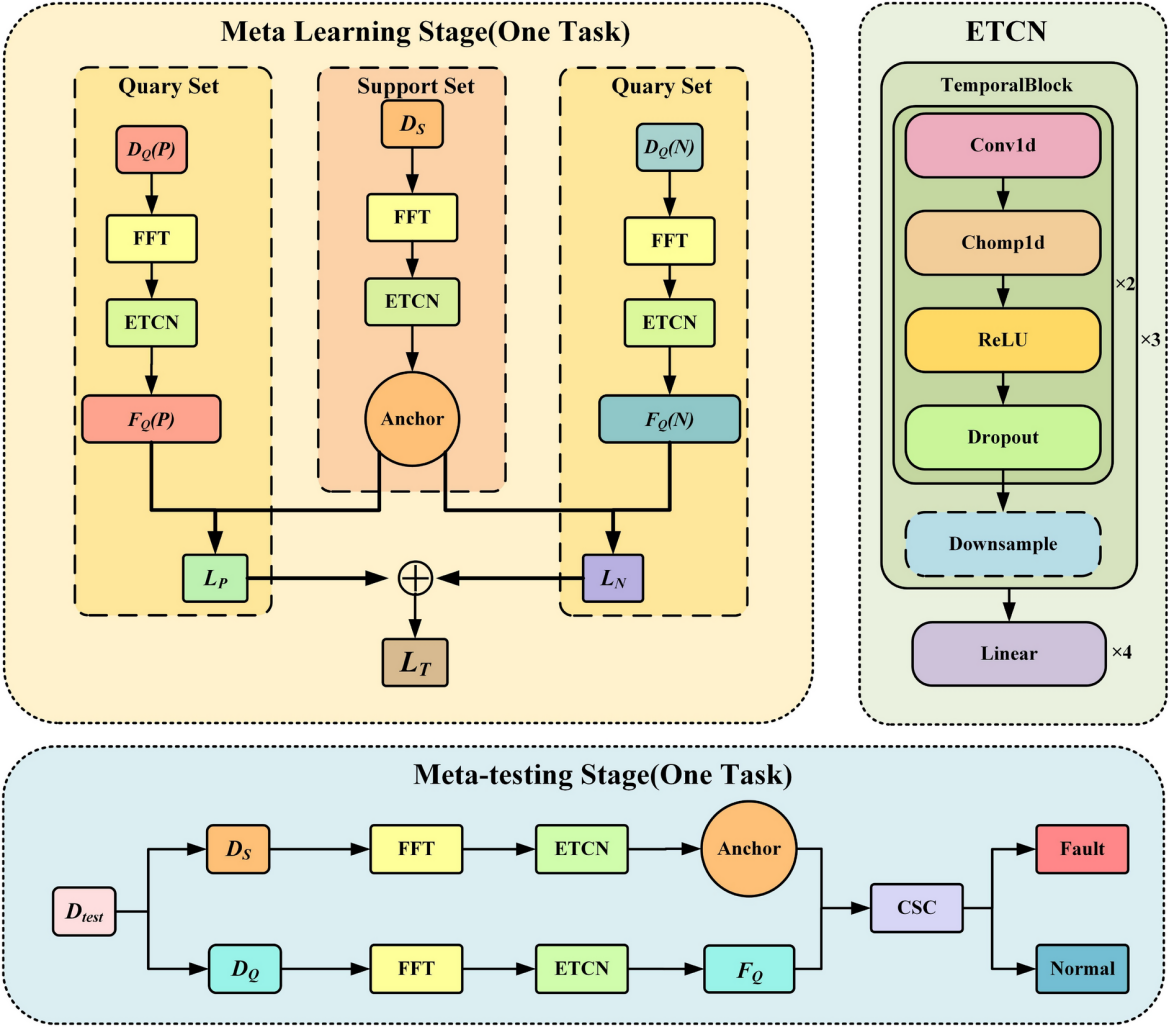
The overall framework of TCPN. The meta learning phase (single task) is a single task of our model training. In a single task, the support set data is transformed into spatial feature anchors after Fourier transform and ETCN, and the positive and negative samples in the query set are compared with the anchors after feature extraction to return the loss. There are three timing blocks in ETCN, and the second timing block does not include subsampling, so it is represented by dotted lines in the figure. In the meta learning test phase, the query set features and anchor points are used to measure and classify in CSC.

In this investigation, we introduce a novel few-shot learning network architecture termed TCPN. The training of this network is grounded in the prototype network paradigm. Building upon this foundation, we have innovatively incorporated Fourier transform, an Enhanced Temporal Convolutional Network (ETCN), and a ContrastSim classifier (CSC) to augment the performance of our neural network. The network learns the characteristics of both normal and fault-bearing signals from few-shot datasets, updating the network’s weights to achieve a high level of generalization. The architecture of the network is depicted in Fig.2.

Our network is modularized into three principal components:

i) The Fourier Transform Module. Here, we apply Fourier transform to convert time-domain normal or fault-bearing signals into frequency-domain signals, retaining only half for input into the feature extractor.
ii) The Enhanced Temporal Convolutional Network (ETCN). We utilize the Temporal Convolutional Network as the fundamental framework, refining its partial structure through the strategic application of causal convolution modules and downsampling to optimize its suitability for bearing signal convolution. Its primary role is to transform the input frequency-domain bearing signal into feature space points for subsequent classification by the input classifier.
iii) The Contrastive Learning Classifier. This component measures the similarity between sample feature points in the feature space and feature-invariant points. This similarity metric is employed for sample classification and to generate loss values.

During each round of single few-shot learning, the process begins by inputting the time-domain signal of the bearing into our ETCN feature extractor via Fourier transform, reducing the data dimensionality from (*n*, 1, 512) to (*n*, 1, 16) spatial feature points. The feature points derived from the support set via the extractor serve as anchor points within our feature space. The comparative learning classifier calculates the similarity measure between the feature points of the query set and these anchor points. This measure guides the updating of network weights through contrastive learning, simultaneously classifying bearing signals and returning the loss from comparative learning to refine the network weights. This iterative process culminates in the optimization of our TCPN model after numerous few-shot training rounds. For testing, the similarity of query sets within the test set is measured using the aforementioned pre-trained model, followed by the classification of few-shot bearing signal data based on these measurement outcomes.

### Detailed description of methods

#### Fourier transform

In the domain of fault detection, the majority of sensors capture time-domain signals; however, the indicative features of a fault typically manifest within a distinct frequency spectrum. Consequently, the transformation of time-domain signals to frequency-domain signals is of paramount importance. To process the discrete sampling points that serve as input to our model, we employ the Discrete Fourier Transform (DFT), a method that illustrates how any periodic signal can be decomposed into a sum of sine and/or cosine waves of varying frequencies. For non-periodic signals, analysis is facilitated by their expansion into periodic equivalents. The mathematical representation of the DFT is provided in Eq. (1)

1 $$\begin{aligned}&X\left[ k \right] =\sum _{N-1}^{n=0} x\left[ n \right] *e^{-\frac{i2\pi }{N}*kn} \end{aligned}$$

where *X*[*k*] represents the transformed frequency domain signal. *x*[*n*] represents the original time domain signal. *N* represents the total number of signal points. *k* represents the index in the frequency domain, ranging from 0 to n-1. *i* represents an imaginary unit.

The direct computational complexity of the Discrete Fourier Transform (DFT) is *O*(*n*2), but this can be significantly reduced to $O(n*log(n))$ through the application of the Fast Fourier Transform (FFT) algorithm. The underlying principle of the FFT algorithm is fundamentally analogous to that of the DFT, yet it markedly enhances the computational speed. The basic principle of FFT is to use the symmetry and periodicity of DFT to reduce the amount of calculation. It decomposes a DFT problem into several smaller DFT problems, and uses the symmetry and periodicity of DFT to reduce repeated calculations. This process can be represented by butterfly operation, which is the basic computing unit in FFT algorithm. Eqs. (2) and 3 define the formula for butterfly operation

2 $$\begin{aligned}&X\left[ k \right] = x_{0} \left[ k\right] *e^{-\frac{i2\pi }{N}*kn} +x_{e} \left[ k \right] \end{aligned}$$

3 $$\begin{aligned}&X\left[ \frac{k+N}{2} \right] = -x_{0} \left[ k\right] *e^{-\frac{i2\pi }{N}*kn} +x_{e} \left[ k \right] \end{aligned}$$

where $x_{e}[k]$ and $x_{0}[k]$ represent even and odd indexed input signals, respectively. *X*[*k*] and $X[k+n/2]$ represent the corresponding output signals. Using FFT, the original time-domain data is transformed into the frequency domain and subsequently fed into a feature extractor for mapping, which can yield a more efficient result.

#### Enhanced temporal convolutional network

To address the challenge of feature extraction from bearing signals, this module was developed based on the Temporal Convolutional Network (TCN). The TCN is a specialized type of convolutional neural network tailored for time series prediction, capable of effectively capturing long-term dependencies within time series data due to its distinctive causal convolution architecture. In contrast to traditional Recurrent Neural Networks (RNNs) and Long-Short Term Memory networks (LSTMs), the TCN demonstrates enhanced computational efficiency in handling extended sequences and mitigates the issue of vanishing gradients. Drawing upon the TCN framework, we have crafted a feature extractor for the current study, termed the Enhanced Temporal Revolutionary Network (ETCN). The ETCN comprises three sequential processing blocks and an additional linear mapping module. The architecture of the specific model is delineated in Fig. 2. As illustrated in Fig. 2, the boundary of the subsampling module is represented with a dashed line, indicating that the subsampling step is omitted in the second sequential processing block. Each of these sequential processing blocks incorporates two causal convolution layers.Suppose $x_t$ is an element in the time series, $h_k$ is the k-th coefficient of the convolution kernel, and the result of one-dimensional convolution $y_t$ is defined by Eq. (4)

4 $$\begin{aligned} y_{t} = (x*h)_{t} = \sum _{k=0}^{K-1} h_{k}*x_{t-k} \end{aligned}$$

where *K* represents the size of convolution kernel, $*$ represents convolution operation, and $x_{t-k}=0$ holds for all $t-k<0$.

In each causal convolution, a one-dimensional convolutional neural network is used to extract features, and then chomp1 d is used to intercept the time series to ensure that the zero padding before the original time series is removed, so that the output only contains the actual data corresponding to the input sequence.The chomp1 d operation can be defined by Eq. (5)

5 $$\begin{aligned} Chomp1d(y_{t})&= y_{t} \quad for\quad t \ge chompsize \end{aligned}$$

where *chompsize* represents the number of zero filled elements removed from the convolution output. Finally, a single causal convolution is achieved through the relu activation function and the dropout process. The whole process of single causal convolution can be defined by Eq. (6)

6 $$\begin{aligned} z_{t}&= ReLU(Dropout(Chomp1d(x*h))) \end{aligned}$$

where $z_t$ represents the output after a one-dimensional convolution, Chomp1 d interception, ReLU activation and dropout processing. To sum up, the whole timing processing block can be defined by Eqs. (7) and (8)

7 $$\begin{aligned} z_{t}^{(1)}&= ReLU(Dropout(Chomp1d(x*h^{(1)}))) \end{aligned}$$

8 $$\begin{aligned} z_{t}^{(2)}&= ReLU(Dropout(Chomp1d(z_{t}^{(1)}*h^{(2)}))) \end{aligned}$$

where $h^{(1)}$ and $h^{(2)}$ represent convolution kernels of two causal convolutions. Downsampling can be realized by pooling, but there is no downsampling in the second sequential processing block. The downsampling in the first and third sequential processing blocks can be defined by Eq. (9)

9 $$\begin{aligned} \bar{z} _t = Pool(z_t) \end{aligned}$$

where *Pool* represents the pool operation and $\bar{z} _t$ represents the sequence after down sampling. After the processing of three time series blocks, the preliminary characteristics of time series signals are obtained. In order to further meet the calculation of feature space similarity, our research design uses a linear layer to map features from (*n*, 1, 512) to (*n*, 1, 16).This linear mapping layer adjusts the correct input for the subsequent classifiers of the model.The whole linear mapping layer has four layers. The first layer is used to map the feature dimension from (*n*, 1, 512) to (*n*, 1, 128), the second layer is used to map the feature dimension from (*n*, 1, 128) to (*n*, 1, 64), the third layer is used to map the feature dimension from (*n*, 1, 64) to (*n*, 1, 32), and the fourth layer is used to map the feature dimension from (*n*, 1, 32) to (*n*, 1, 16). Their overall principle can be defined by Eq. (10)

10 $$\begin{aligned} f&= W*z+b \end{aligned}$$

where *f* represents the mapped eigenvector, *W* represents the weight matrix, *z* represents the eigenvector processed by the timing processing block, and *b* represents the offset term.Specifically, when the function maps a tensor with dimension (*n*, 1, *p*) to a tensor with dimension (*n*, 1, *q*), the dimension of *W* is (*q*, *p*), and the dimension of *b* is (*q*, 1, 1). At this point, the feature extraction process of the entire ETCN we designed is over. The original high-dimensional time series data points are mapped to anchor points or sample points in the feature space that can be used for similarity measurement.

#### ContrastSim classifier and ContrastSim loss

For the feature space points generated by ETCN, we start from the principle of classification, abandon the previous classification method of distance measurement of prototype network, and use similarity to determine the difference between data anchor points and data sample points. To determine whether the bearing signal is normal or fault in our experiment. In the past research, the three commonly used distances are Euclidean distance, Manhattan distance and Chebyshev distance. Suppose there are *n*-dimensional vector $A(x_{1}, x_{2},\cdots ,x_{n})$ and *n*-dimensional vector $B(y_{1}, y_{2},\cdots ,y_{n})$. The calculation of these distance metrics can be defined as Eqs. (11), (12) and (13)

11 $$\begin{aligned} d_E&= \sqrt{(x_1-y_1)^2+(x_2-y_2)^2+\cdots + (x_n-y_n)^2} \end{aligned}$$

12 $$\begin{aligned} d_M&= \left| x_1-y_1 \right| +\left| x_2-y_2 \right| +\cdots +\left| x_n-y_n \right| \end{aligned}$$

13 $$\begin{aligned} d_C&= \max (\left| x_1-y_1 \right| ,\left| x_2-y_2 \right| ,\cdots +\left| x_n-y_n \right| ) \end{aligned}$$

where $d_E$ represents Euclidean distance, $d_M$ represents Manhattan distance, and $d_C$ represents Chebyshev distance. However, the calculation of distance is not enough to accurately measure the accurate classification of data points. In our research, we propose to use cosine similarity to replace the distance metric in the prototype network and design our ContractSim Classifier (CSC). In a single few-shot learning task, we use the entire support set as the anchor of the feature space, and use the positive or negative samples of the query set for similarity measurement. Suppose that the support set *A* is $(A_1,\cdots ,A_j,\cdots ,A_k)$, where $A_j$ is $(x_{1}, x_{2},\cdots ,x_{n})$, and the single sample B in the query set is $(y_{1}, y_{2},\cdots ,y_{n})$. The calculation of our ContractSim can be defined as Eqs. (14) and (15)

14 $$\begin{aligned} \cos (A_j,B_j)&= \frac{ {\textstyle \sum _{i = 1}^{n}x_i*y_i} }{\sqrt{ {\textstyle \sum _{i = 1}^{n}x_{i}^{2} } }\sqrt{ {\textstyle \sum _{i = 1}^{n}y_{i}^{2} } } } \end{aligned}$$

15 $$\begin{aligned} ContractSim(A,B)&= \frac{ {\textstyle \sum _{j = 1}^{k}\cos (A_j,B)} }{k} \end{aligned}$$

where $\sum _{i = 1}^{n}x_i*y_i$ represents the dot product of vectors $A_j$ and *B*, and $\sum _{i = 1}^{n}x_{i}^{2}$ and $\sum _{i = 1}^{n}y_{i}^{2}$ represent the Euclidean norms of $A_j$ and *B*, respectively. *k* represents the number of samples in the support set. Through the method of cosine similarity in the above, we designed the loss function ContractSim loss for our classifier CSC. Because the cosine similarity range is from −1 to 1, it cannot be directly used for return loss.Considering that the loss value of the same category of samples should decrease with the training, and the loss value of different categories should increase with the training. We design ContractSim loss as Eqs. (16), (17) and (18)

16 $$\begin{aligned} L_P&= ContractSim(f(P),Anchor) \end{aligned}$$

17 $$\begin{aligned} L_N&= ContractSim(f(N),Anchor) \end{aligned}$$

18 $$\begin{aligned} L_T&= 2+L_P-L_N \end{aligned}$$

where $L_P$ represents the training loss of positive samples in the prototype network, and $L_N$ represents the training loss of negative samples in the prototype network. *f*(*x*) represents the feature extractor ETCN. *Anchor* represents the mapping point of the support set in the feature space, that is, the anchor point. $L_T$ is ContractSim loss. Our ETCN updates the weight in each few-shot learning task according to the back propagation of contractsim loss. Then the pre-training model of TCPN is generated.

### Few-shot training and testing of TCPN

We divided the support set and query set for few-shot learning in the training set and test set, and constructed our 2-way-k-shot prototype network for meta learning.

**Algorithm 1:**
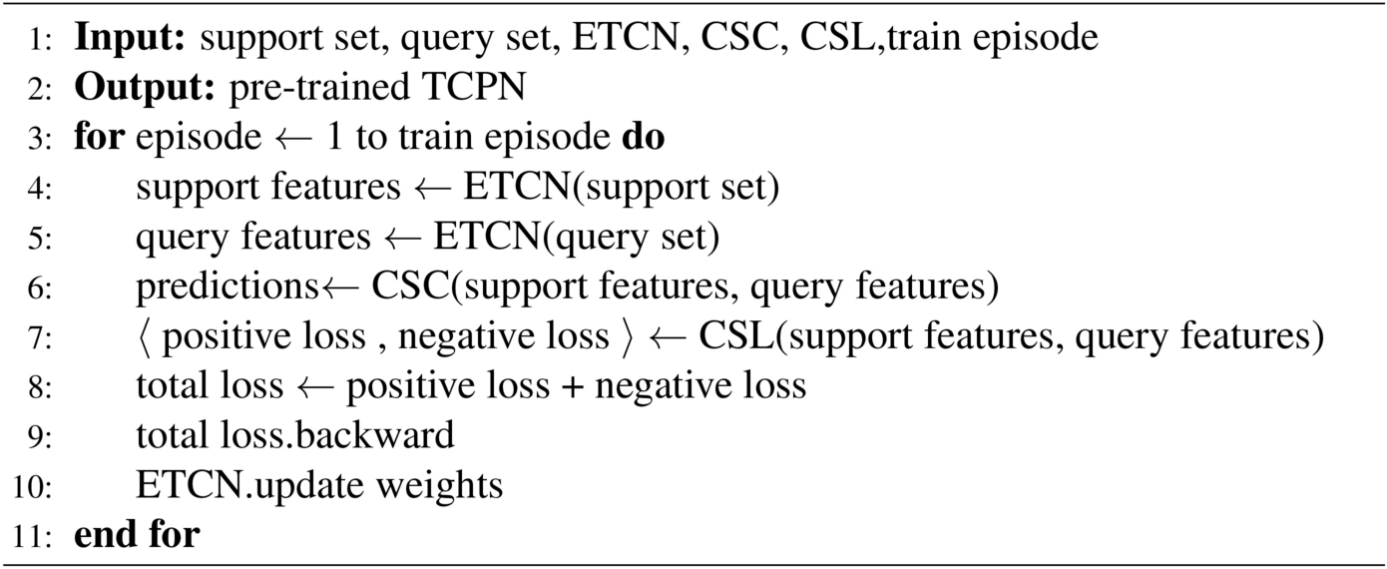
Training Process with TCPN.

**Algorithm 2:**
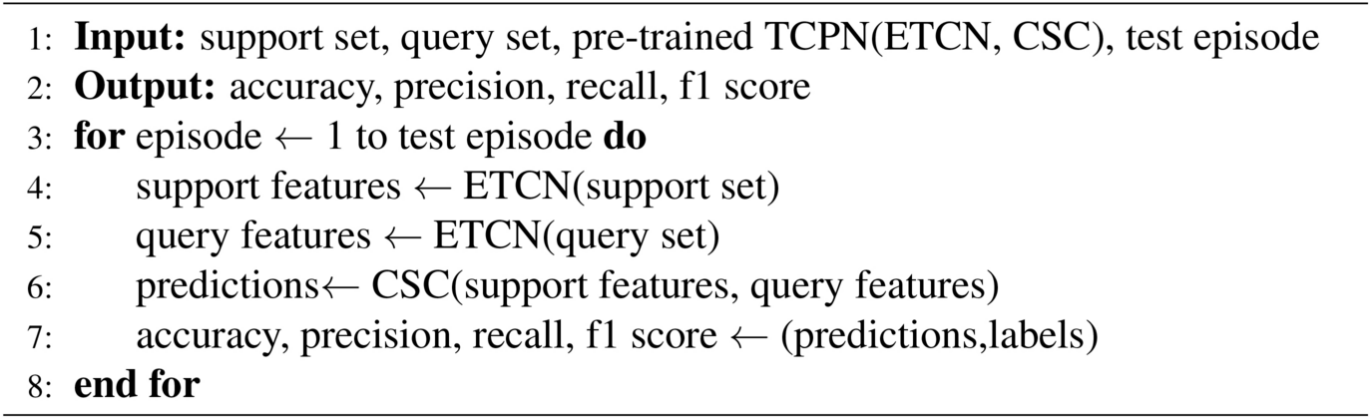
Test Process with TCPN.

The whole TCPN network training process is shown in algorithm 1. First, support set and query set are used as input data samples for training. At the end of the whole training process, the TCPN model of pre training is finally output. In each round of few-shot learning task, K samples in the support set are firstly Fourier transformed to highlight their frequency domain characteristics. Then the frequency domain feature is convoluted in a series of time series in the etcn feature extractor, and becomes the feature of the low latitude feature space. Since this feature is derived from the support set and can be used as the Classification Benchmark, it should be used as the anchor point of the feature space in a single training task. In the same few-shot learning task, the positive and negative samples in the query set use the same feature mapping method to get the sample characteristics of the query set. The positive sample feature in the query set is compared with the anchor in the feature space to return the positive sample loss, while the negative sample feature is compared with the anchor in the feature space to return the negative sample loss. The two losses are calculated according to formula 18 to obtain the final total loss. The total loss will be returned to the feature extractor for learning to adjust the weight of the model, so that the new round of few-shot task has better effect. After all the few-shot training tasks, our TCPN model finally becomes a pre training model, ready to enter the test phase to complete the effect test of the model.

The test of the pre trained TCPN model is shown in algorithm 2. Since the TCPN model is adjusted without returning the loss of comparative learning, the few-shot task in the test phase is also reduced. In each round of few-shot task test, the K samples of the support set are first transformed into frequency domain signals by fast Fourier transform. After feature extraction of ETCN, it is mapped to anchor points in the feature space of a single few-shot test task. The two unknown samples in the query set are mapped to the feature space through fast Fourier transform and etcn feature extractor, and become two vectors belonging to the feature space. In the CSC designed by us, the similarity measure is obtained between the eigenvectors of two unknown samples and the anchor point according to formula 15. According to the similarity measurement results, CSC will classify the samples in the query set. Finally, the classification results will be compared with the real labels, and the final effect index of the model will be obtained.

The training of the whole TCPN model is set with multiple tasks, aiming to make full use of the support set and query set in each round to get effective feedback for the model. This will help the model to adjust the weight in a more accurate direction. The test task of TCPN model aims to reduce the contingency of a single few-shot model test task and make the final result statistically significant.

## Experiments

### Dataset and evaluation metrics

**Dataset.**To validate the efficacy of the proposed method for the diagnosis of bearing faults, we used four renowned public datasets: the Case Western Reserve University dataset (CWRU)^54^, the Jiangnan University bearing dataset(JNU-BDS)^55^, University of Ottawa Bearing Dataset(Ottawa)^56^, and the The Society for Machinery Failure Prevention Technology (MFPT) bearings dataset^57^. The CWRU dataset encompasses four distinct categories of bearing fault data, including inner ring fault, outer ring fault, rolling element fault, and normal condition, with varying fault severities. The dataset predominantly features a sampling frequency of 12 kHz, with a portion of the data available at alternative frequencies. It is further categorized by fault diameter, such as 0.1778 mm, 0.3556 mm, and 0.5334 mm. The Jiangnan University bearing dataset primarily includes data on inner ring failure, outer ring failure, rolling element failure, and normal operating conditions. The vibration signals are typically provided in the CSV or.mat file format, with a standard sampling frequency of approximately 20 kHz. This data set is also classified by fault diameter, including 0.1, 0.2, and 0.3 mm, among others. The Ottawa bearing dataset covers the normal state, inner ring fault, outer ring fault, and rolling element fault conditions. The vibration signals are provided in.mat file format, with a general sampling frequency of 25600 Hz. It includes samples with varying degree of fault, such as 0.18 mm, 0.36 mm, and 0.53 mm. The MFPT bearing dataset comprises vibration signals from a ball bearing under normal operation and various fault conditions, including different load and speed scenarios. It encompasses normal states and varying degrees of inner and outer ring faults, with three normal states (270 pounds load, 25 Hz speed) and seven fault states (varying load, 25 Hz speed). The sampling rates are 97656 and 48828 Hz, respectively, and the data are provided in.mat file format, suitable for bearing fault diagnosis research. For these diverse datasets, our study applied uniform data processing methods. Initially, the data were labeled according to their normal and fault categories. Subsequently, 70$\%$ of the data was allocated to the training set, while the remaining 30$\%$ were designated for the test set. Within the training and test sets, our research constructed single tasks with five normal bearing signals as the support set and one normal bearing signal along with one fault signal as the query set. This approach resulted in the creation of 2000 training tasks and 100 test tasks for each dataset. Consequently, we have treated these fault datasets as few-shot learning datasets.

**Evaluation Metrics.** In the realm of machine learning, four standard performance metrics are employed to assess the efficacy of a model: Accuracy, Precision, Recall, and the F1 score.The results of the four evaluation indexes in our research results have retained four decimal places. Accuracy represents the proportion of correct predictions made by the model, thereby serving as an indicator of the model’s overall predictive accuracy.Precision, on the other hand, is defined as the fraction of predicted positive samples that are indeed positive, providing a measure of the model’s precision in identifying positive instances.The recall rate quantifies the proportion of actual positive samples that are correctly identified by the model, thus reflecting the model’s capacity to detect positive cases.The F1 score is computed as the harmonic mean of precision and recall, offering a comprehensive measure of the model’s balance between precision and recall. The calculation of these four metrics is defined as Eqs. (19), (20), (21) and (22)

19 $$\begin{aligned}&{Accuracy} = \frac{TP+TN}{TP+TN+FP+FN} \end{aligned}$$

20 $$\begin{aligned}&{Precision}=\frac{TP}{TP+FP} \end{aligned}$$

21 $$\begin{aligned}&{Recall}=\frac{TP}{TP+FN} \end{aligned}$$

22 $$\begin{aligned}&{F1 score}=2*\frac{Pr*Re}{Pr+Re} \end{aligned}$$

where *TP* represents the number of samples correctly predicted as positive by the model, *FP* represents the number of samples that are incorrectly predicted as positive by the model, *TN* represents the number of samples correctly predicted as negative by the model and *FN* represents the number of samples that are incorrectly predicted as negative by the model.

Furthermore, we investigate the t-distributed Stochastic Neighbor Embedding (t-SNE) diagram and the Receiver Operating Characteristic (ROC) curve to illustrate our experimental findings. The t-SNE diagram provides a direct visualization of the quality of data clustering, as evidenced by the legend. The ROC curve, on the other hand, is a graphical representation of the relationship between the True Positive Rate (TPR) and the False Positive Rate (FPR). A ROC curve that approaches the upper left corner of the graph indicates superior model performance. Additionally, the Area Under the Curve (AUC) of the ROC is a metric used to assess model performance. The AUC value ranges between 0 and 1, with higher values signifying better model performance.

### Implementation details

**Architecture.** In our study, a uniform data processing methodology is applied to the initial data files across the four datasets. Initially, a script is employed to extract data using a 1024-sample time window and a 1024-sample time step, with normal and fault signals being labeled as “1” and “−1,” respectively. The datasets are partitioned into training and testing subsets at a 7:3 ratio, resulting in a training set dimension of [3150, 1024] and a test set dimension of [1350, 1024].Subsequently, the training set is further divided into support and query sets based on the number of episodes. For instance, in a 5-shot scenario, the support set dimension is [5, 1024], while the query set dimension is [2, 1024]. The same division protocol is applied to the test sets. The input dimension for the model is [n, 1024], where ’n’ represents the number of samples.Following feature extraction by the feature extractor, e.g., the ETCN, the output dimension of the model becomes [n, 1, 16]. This output is then fed into the comparative learning classifier CSC for classification and loss computation, culminating in the pre-training of the model.During the test phase, the model output with the dimension [n, 1, 16] is directly inputted into the classifier for classification purposes, and the model’s performance is evaluated based on four testing metrics.

**Training.** The model employed in our research utilizes the Adam optimizer, with a learning rate of 0.00005. The beta parameters for the optimizer are set at 0.5 and 0.9, respectively. For the loss function, we have developed a custom-designed function tailored for few-shot comparative learning, as detailed in Chapter III of this manuscript. The entire training process was executed on an NVIDIA RTX 4080S GPU with 16GB of video memory, encompassing a total of 2000 few-shot training episodes and 100 few-shot testing episodes. The model of this research ran and learned in Pycharm with version 2024.1.4 under Windows 11 operating system. The interpreter used for all related code is Python 3.8.10.

### Results of K-shot experiments

**Fig. 3:**
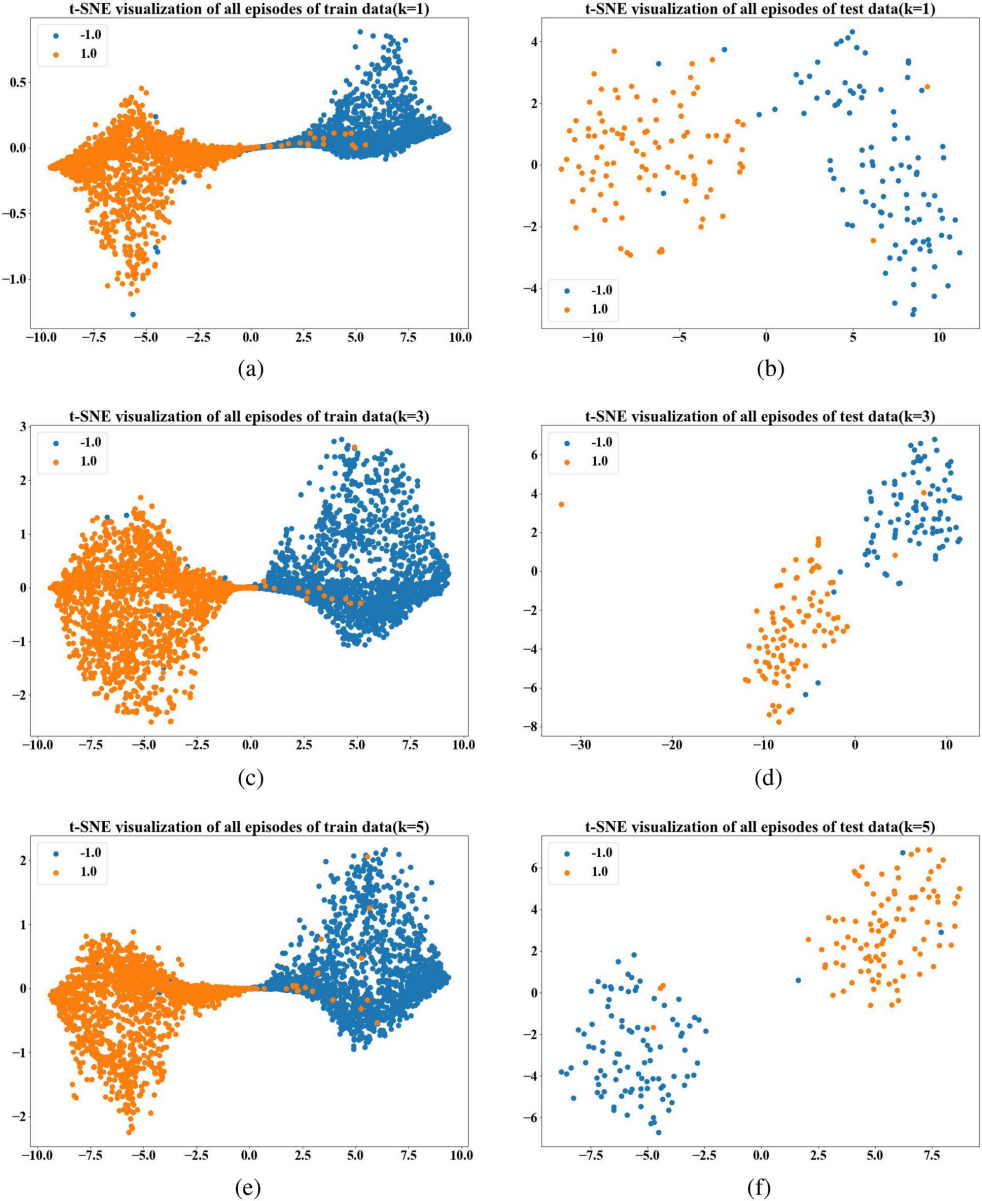
(**a**) is the classification of the model on the training set when k=1. (**c**) is the classification of the model on the training set when k=3. (**e**) Is the classification of the model on the training set when k=5. (**b**) Is the classification of the model on the test set when k=1. (**d**) Is the classification of the model on the test set when k=3. (**f**) Is the classification of the model on the test set when k=5.

In the context of few-shot learning for deep learning models, our investigation is dedicated to identifying the optimal representation of data features within the feature space. Our approach involves manipulating the number of samples in the support set to pinpoint the location of the data anchor. The role of the data anchor is to identify a feature-invariant point for these samples in the feature space, facilitating the learning process for the deep learning model to differentiate between normal and abnormal bearing signals.

**Table 1:** The contents in the table are the experiments on four datasets when k=1, k=2, k=3, k=4, k=5 respectively.The evaluation indicators are accuracy,precision,recall and F1 Score. The experimental results retain the significant digits for further comparison.

Dataset	K	Accuracy	Recall	Precision	F1 Score
CWRU	1	0.8000	0.9000	0.7500	0.8182
	2	0.8500	0.9100	0.8125	0.8585
	3	0.8500	0.9300	0.8017	0.8611
	4	0.8500	0.9400	0.7966	0.8624
	5	**0.9300**	**0.9400**	**0.9216**	**0.9307**
Ottawa	1	0.8100	0.8000	0.8163	0.8081
	2	0.8500	0.8600	0.8431	0.8515
	3	0.8300	0.8300	0.8300	0.8300
	4	0.8800	0.8900	0.8725	0.8812
	5	**0.8950**	**0.8900**	**0.8990**	**0.8945**
JNU-BDS	1	0.7900	0.7900	0.7900	0.7900
	2	0.7900	0.8000	0.7843	0.7921
	3	0.8300	0.8300	0.8300	0.8300
	4	0.8200	0.8200	0.8200	0.8200
	5	**0.8650**	**0.8700**	**0.8614**	**0.8657**
MFPT	1	0.8150	0.8200	0.8119	0.8159
	2	0.8100	0.8100	0.8100	0.8100
	3	0.8600	0.8400	0.8750	0.8571
	4	0.7800	0.7800	0.7800	0.7800
	5	**0.8750**	**0.8600**	**0.8866**	**0.8731**

**Fig. 4:**
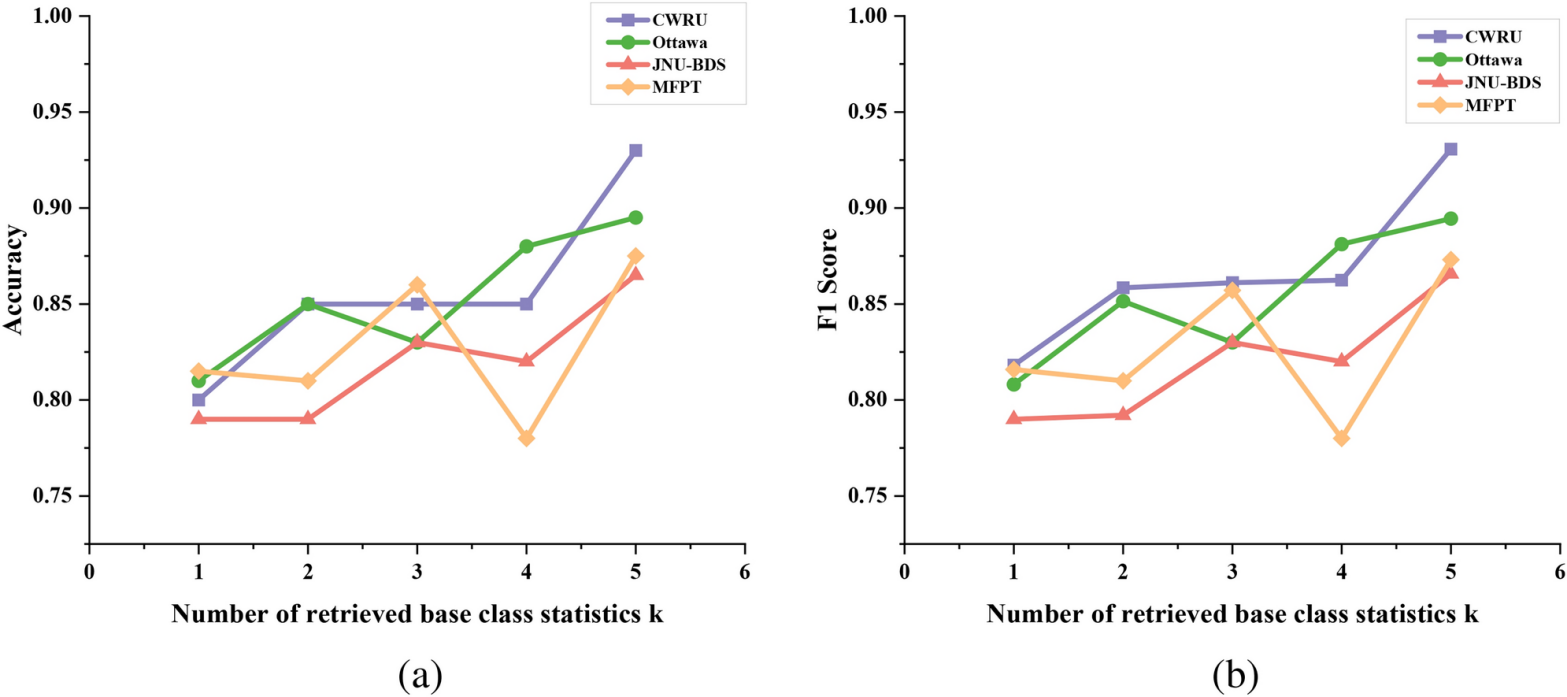
(**a**) is a line chart showing the change in the accuracy of the model over the four data sets when k is from 1 to 5. (**b**) is the line chart of F1 score change of the model on four data sets when k is from 1 to 5.

To visualize the classification capabilities of our model during both training and testing phases, we employ t-SNE diagrams. Figure 3 illustrates the model’s classification efficacy as the parameter K varies from 1 to 5 during the training phase, with subfigures (a),(c) and (e) depicting this variation. Conversely, subfigures (b),(d) and (f) in the same figure represent the model’s classification efficacy during the test phase as parameter K changes from 1 to 5. Table 1 provides a quantitative analysis of our model’s fault diagnosis performance under varying values of parameter K, offering a precise depiction of the changes observed.Additionally, to render the experimental outcomes more intuitive, Fig. 4 presents a line chart that visualizes the accuracy and F1 score data.

The k-shot experimental results indicate that our few-shot model achieves its highest classification efficacy when k is set to 5. This finding further underscores that within the few-shot domain, an increase in the number of samples more accurately represents the data anchor’s position in the feature space within the prototype network, thereby enhancing the model’s ability to discern between normal and fault-bearing signals.

### Comparison with advanced models

**Fig. 5:**
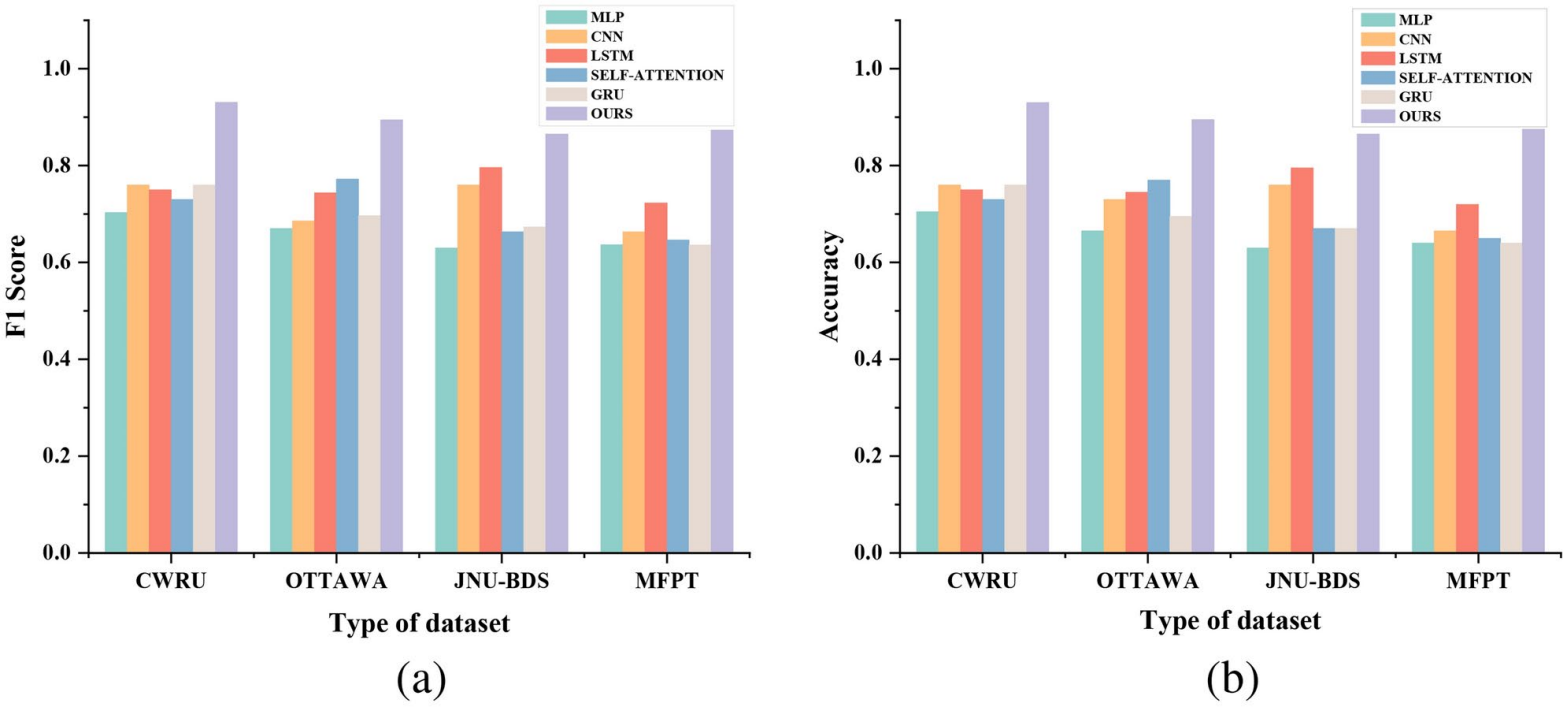
(**a**) is a bar graph of the accuracy of six model experiments including our model on four datasets. (**b**) is a bar graph of F1 score of six model experiments including our model on four datasets.

To underscore the efficacy of our proposed model, we conducted a series of comparative experiments involving six models, including ours, across four distinct datasets. The models selected for comparison were Multilayer Perceptrons (MLP), Convolutional Neural Networks (CNN), Multi-Head Self-Attention, Gated Recurrent Units (GRU), and Long Short-Term Memory networks (LSTM). These architectures have demonstrated significant utility in both deep learning and fault detection domains. Within the few-shot learning paradigm, we employed these models as feature extractors for the purpose of comparative analysis, thereby elucidating the superior attributes of our model framework. All experiments were conducted under the condition of k=5, and the results across the four datasets are presented in Table 2. Figure 5 provides a bar graph visualization of these outcomes, offering a intuitive representation of our model’s enhanced performance.

**Table 2:** The contents in the table are the comparative experiments of six common models including our model on four datasets. The evaluation indicators are accuracy, precision, recall, F1 Score. The experimental results retain the significant digits for further comparison.

Dataset	Modal	Accuracy	Recall	Precision	F1 Score
CWRU	MLP	0.7050	0.7000	0.7071	0.7035
	CNN	0.7600	0.7600	0.7600	0.7600
	Self-Attention	0.7300	0.7500	0.7212	0.7353
	LSTM	0.7500	0.7500	0.7500	0.7500
	GRU	0.7600	0.7600	0.7600	0.7600
	**ours**	**0.9300**	**0.9400**	**0.9216**	**0.9307**
Ottawa	MLP	0.6650	0.6700	0.6634	0.6667
	CNN	0.7300	0.7400	0.7255	0.6866
	Self-Attention	0.7700	0.7800	0.7647	0.7723
	LSTM	0.7450	0.7400	0.7475	0.7437
	GRU	0.6950	0.7000	0.6931	0.6965
	**ours**	**0.8950**	**0.8900**	**0.8990**	**0.8945**
JNU-BDS	MLP	0.6300	0.6300	0.6300	0.6300
	CNN	0.7600	0.7600	0.7600	0.7600
	Self-Attention	0.6700	0.6500	0.6771	0.6633
	LSTM	0.7950	0.8000	0.7921	0.7960
	GRU	0.6700	0.6800	0.6667	0.6733
	**ours**	**0.8650**	**0.8700**	**0.8614**	**0.8657**
MFPT	MLP	0.6400	0.6300	0.6429	0.6364
	CNN	0.6650	0.6600	0.6667	0.6633
	Self-Attention	0.6500	0.6400	0.6531	0.6465
	LSTM	0.7200	0.7300	0.7157	0.7228
	GRU	0.6400	0.6300	0.6429	0.6364
	**ours**	**0.8750**	**0.8600**	**0.8866**	**0.8731**

The comparative results indicate that the performance of the other five models varies across different datasets. For instance, the CNN exhibited suboptimal performance on the MFPT dataset, yet achieved an accuracy of approximately 0.75 on the other three datasets. As a foundational neural network, the Multi-Sensor network did not perform as effectively as other feature extractors. The Multi-Head Self-Attention mechanism yielded the best results on the Canadian dataset, with an accuracy and F1 score of 0.77 each. As a fundamental variant of TCN, the LSTM demonstrated consistent performance across all four datasets, with accuracy and F1 scores remaining above 0.7. The GRU’s performance was akin to that of the CNN, with notably superior results on the CWRU dataset compared to the other three. However, the GRU did not exhibit strong generalization across datasets. Ultimately, our model achieved the most favorable outcomes on the CWRU dataset, with accuracy and F1 scores 0.17 higher than those of both the CNN and GRU. Across the remaining three datasets, it also maintained stable and superior accuracy, recall, precision, and F1 scores.

**Fig. 6:**
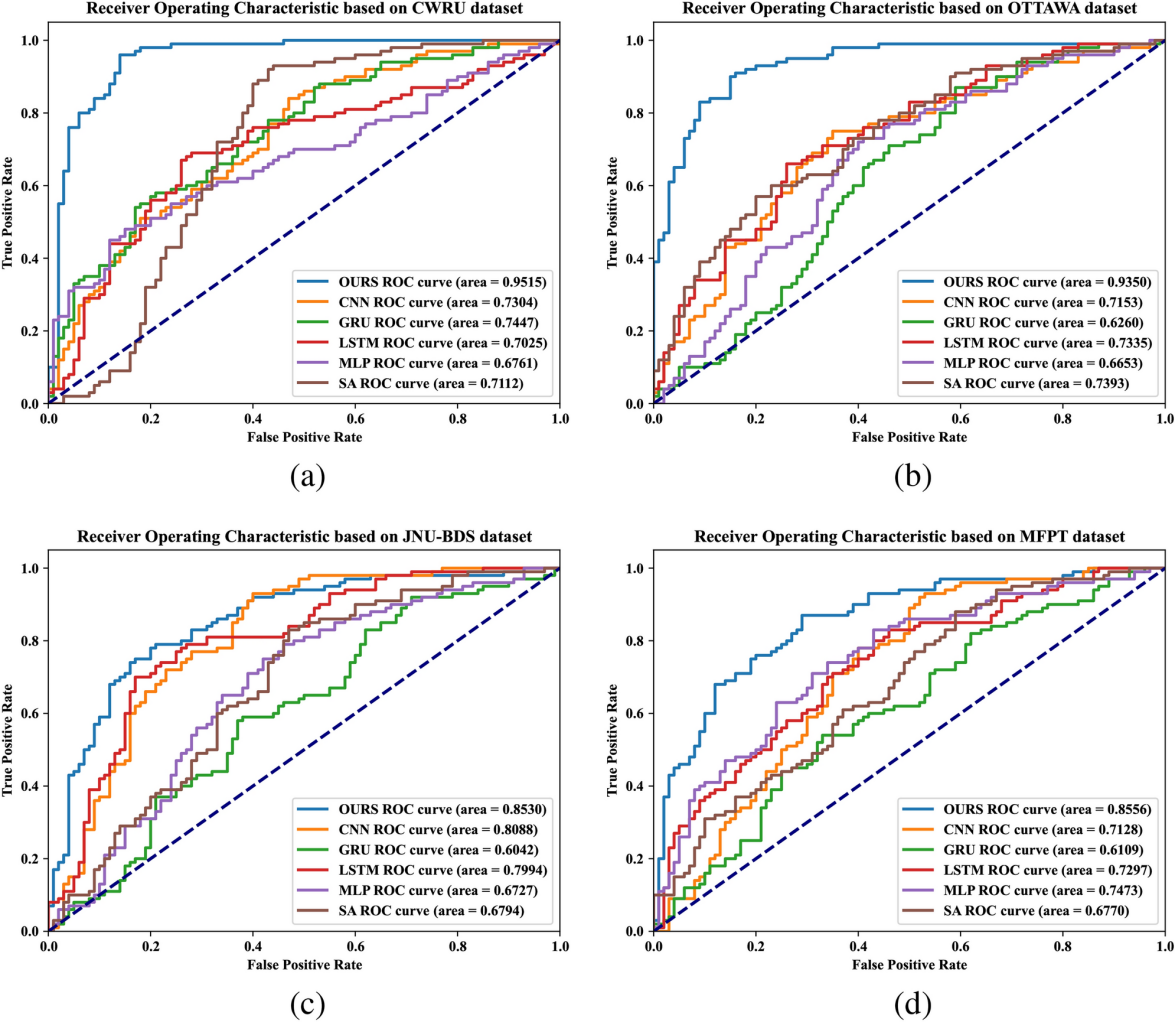
(**a**) is the ROC diagram of six model experiments including our model on the CWRU dataset. (**b**) is the ROC diagram of six model experiments including our model on the jnu-bds dataset. (**c**) is the ROC diagram of six model experiments including our model on Ottawa dataset. (**d**) is the ROC diagram of six model experiments including our model on the MFPT dataset.

Furthermore, on the Receiver Operating Characteristic (ROC) curves constructed using True Positive Rate (TPR) and False Positive Rate (FPR), our model TCPN demonstrated the greatest capability in distinguishing between positive and negative examples. The specific visualization is provided in Fig. 6. The Area Under the Curve (AUC) values for our model across the four datasets were the highest, all exceeding 0.86, indicating a high level of performance. This further validates our model’s proficiency in handling various thresholds while maintaining excellence in the original four metrics.

The results of the model comparison experiments reveal that, despite the variable performance of different feature extractors across each dataset, the time-series convolutional neural network-based improved feature extractor, ETCN, proposed by us, consistently yielded the most stable and effective feature extraction results across all datasets.

### Comparison with distance classifier

The core concept underpinning our proposed CSC classifier is the substitution of the traditional distance metric in prototype networks with cosine similarity. Given that the classifier constitutes the backbone of the neural network, it is deemed imprudent to conduct ablation studies. Instead, we have conducted a comparative experiment focusing on our proposed contrastive learning classifier. The comparison includes the three most frequently utilized distance metrics in prototype networks: Euclidean, Manhattan, and Chebyshev distances. We evaluated these four distance metrics, including our classifier, across four datasets under the condition of k=5, and the results are presented in the Table 3.

**Table 3:** The contents in the table are the comparative experiments of four classifiers including our classifier on four datasets. The evaluation indicators are accuracy, precision, recall and F1 Score. The experimental results retain the significant digits for further comparison.

Dataset	Classifier	Accuracy	Recall	Precision	F1 Score
CWRU	Euclidean	0.8950	0.8400	0.9438	0.8889
	Manhattan	0.9000	0.8600	0.9348	0.8958
	Chebyshev	0.8650	0.8000	0.9195	0.8556
	**ours**	**0.9300**	**0.9400**	**0.9216**	**0.9307**
Ottawa	Euclidean	0.8650	0.8700	0.8614	0.8657
	Manhattan	0.8550	0.8000	0.8989	0.8466
	Chebyshev	0.8250	0.8600	0.8037	0.8309
	**ours**	**0.8950**	**0.8900**	**0.8990**	**0.8945**
JNU-BDS	Euclidean	0.7650	0.8600	0.7227	0.7854
	Manhattan	0.7300	0.8200	0.6949	0.7523
	Chebyshev	0.7500	0.8500	0.7083	0.7727
	**ours**	**0.8650**	**0.8700**	**0.8614**	**0.8657**
MFPT	Euclidean	0.7900	0.8000	0.7843	0.7921
	Manhattan	0.8200	0.8500	0.8019	0.8252
	Chebyshev	0.7000	0.7600	0.6786	0.7170
	**ours**	**0.8750**	**0.8600**	**0.8866**	**0.8731**

The experimental outcomes indicate that cosine similarity outperforms the three conventional distance metrics as the foundation of the classifier. On the CWRU dataset, our contrastive learning classifier achieved an accuracy 0.03 higher than the optimal Manhattan distance classifier and 0.65 higher than the least effective Chebyshev distance classifier. With respect to the F1 score, our classifier surpassed the best Manhattan distance classifier by approximately 0.035 and the worst Chebyshev classifier by about 0.075. Similar improvements were observed across the other datasets, thereby substantiating our approach. The essence of classification lies in the differentiation and categorization of data, and the fundamental element that bridges these two aspects is similarity.

### Ablation study

Our model introduces an innovative integration of the fast Fourier transform for signal processing, the feature extractor ETCN, and the contrastive learning classifier, CSC. Having established the superiority of the contrastive learning classifier within our network through classifier comparison experiments in the preceding chapter, and recognizing the imprudence of conducting ablation experiments on the classifier, we elected to focus our ablation studies on the modules of fast Fourier transform and the feature extractor ETCN. The ablation experiments in this study were conducted on these two modules, with the experiments carried out under the condition of k=5 to maintain the integrity of the model’s experimental effect. Each module’s experiment was based on four datasets to ensure the precision and validity of the outcomes. Table 4 presents the results when the Fourier transform module was ablated and displays the outcomes when only the ETCN module was removed.

**Table 4:** The contents in the table are the results of ablation experiments on four datasets respectively for the Fourier transform module and ETCN feature extractor module.The evaluation indicators are accuracy,precision,recall and F1 Score.The experimental results retain the significant digits for further comparison.

Dataset	FFT	ETCN	Accuracy	Recall	Precision	F1 Score
CWRU	**-**	**+**	0.5050	0.5800	0.5051	0.5370
	**+**	**-**	0.8600	0.8600	0.8600	0.8600
	**+**	**+**	**0.9300**	**0.9400**	**0.9216**	**0.9307**
Ottawa	**-**	**+**	0.6550	0.6400	0.6598	0.6497
	**+**	**-**	0.6800	0.6800	0.6800	68.0000
	**+**	**+**	**0.8950**	**0.8900**	**0.8990**	**0.8945**
JNU-BDS	**-**	**+**	0.5100	0.5200	0.5098	0.5149
	**+**	**-**	0.8200	0.8200	0.8200	0.8200
	**+**	**+**	**0.8650**	**0.8700**	**0.8614**	**0.8657**
MFPT	**-**	**+**	0.4850	0.4800	0.4848	0.4824
	**+**	**-**	0.6700	0.6700	0.6700	0.6700
	**+**	**+**	**0.8750**	**0.8600**	**0.8866**	**0.8731**

The experimental findings indicate that the removal of the Fourier transform module from the complete model significantly diminishes the classification efficacy across all four datasets. The Ottawa dataset experienced the smallest decline in accuracy, approximately 0.24, whereas the CWRU dataset’s accuracy decreased by about 0.425. The F1 score metric exhibited the most significant drop on the CWRU dataset, around 0.393, and the least decline on the Ottawa bearing dataset, approximately 0.245. These results underscore the necessity of employing Fourier transform to convert signals from the time domain to the frequency domain, thereby effectively accentuating the distinction between fault and normal signals, which in turn facilitates feature extraction by the TCPN model.

Furthermore, the experimental data reveal that when the TCN module is omitted from the model for feature extraction, the classification performance for fault and normal signals on the four datasets is compromised to some extent. The Jiangnan University bearing dataset exhibited the smallest decrease in accuracy, about 0.045, while the Ottawa bearing dataset’s accuracy decreased notably, about 0.215. The F1 score saw the most significant reduction in the Ottawa bearing dataset, approximately 0.214, and the least decrease in the Jiangnan University bearing dataset, about 0.046. This underscores the importance of extracting deep features from bearing signals using the time-series convolutional network, which aids the contrastive learning classifier designed in this study in distinguishing between normal and fault bearing signals.

Synthesizing the results from both the classifier comparison and ablation experiments, our research confirms the efficacy of the three integrated modules across multiple datasets. The degradation in model performance upon the absence of these modules indicates their indispensability. The collaborative action of these three modules is pivotal to the success of this research.

### Discussion

The experimental outcomes indicate that the deep learning network, termed TCPN, which we have developed, exhibits several notable advantages:

- Our few-shot deep learning approach effectively characterizes the data anchor’s position within the feature space. By performing a Fourier transform on the few-shot signals prior to their input into the neural network, we successfully convert the intricate time-domain signals into the frequency domain. This conversion elucidates the frequency components of the signals, thereby enhancing the prominence of fault characteristic frequencies. To reduce data volume while preserving the symmetry of the signal in the frequency domain, our method employs only the first half of the frequency data as network input, significantly diminishing computational load while maintaining data characteristics.
- To align the network’s feature extraction capabilities with the nature of time-series signals, we have enhanced the Temporal Convolutional network(TCN), resulting in a more efficient Temporal Convolutional Network. The TCN excels in extracting features from time-series signals by leveraging dilated convolution layers, which capture long-range dependencies while preserving temporal sequence information. The ETCN employed in our study features a sophisticated architecture, incorporating fully connected layers and causal convolution layers, thereby constituting a more complex and capable network model. Our experimental results demonstrate that ETCN exhibits strong adaptability and generalization when processing both normal and fault-bearing signals.
- In contrast to previous few-shot prototype networks that primarily compute the distance between samples and feature-invariant points to differentiate between normal and abnormal bearing signals, our research introduces an innovative approach. We utilize cosine similarity to construct the Contrastive Similarity Classifier (CSC) and its corresponding loss function. By comparing the similarities between positive and negative samples in the query set and feature-invariant points, the model is trained to classify and pre-train effectively. Subsequent adjustment of the network weights is achieved through back-propagation, fine-tuning the CSC and loss function. Our integrated model has yielded superior results.

Across multiple datasets, the model experiments have revealed that our proposed model, which amalgamates the benefits of Fourier transform, ETCN, and CSC, attains the most favorable experimental outcomes compared to other models. The consistency of these results across various datasets further validates the efficacy of our model and its robust generalization in the domain of fault detection.

## Conclusion

This study introduces a novel and efficacious prototype network architecture, termed TCPN, which is grounded in the principles of few-shot learning. The proposed TCPN innovatively integrates Fourier transform, an enhanced feature extractor based on the Temporal Convolutional Network (ETCN), and a comparative learning classifier, CSC, which operates on cosine similarity. This architecture enables the neural network to effectively discriminate between normal and fault bearing signals without the necessity of increasing the data volume. In the model experimentation phase, we conducted a series of tests using four datasets that are widely employed in fault detection research. The experimental results clearly show that our model is superior to other models in such tasks. The results show that our work is effective in the field of few-shot learning fault detection and has good generalization performance. At the same time, our proposed etcn also has a complex network structure. To ensure the effect of the model while simplifying the model will be an improvement measure of the model. In the future, the research of TCPN model will be committed to integrating into an efficient industrial real-time monitoring system to timely detect bearing faults and make predictive prevention.

## Data Availability

The datasets analyzed during the current study were derived from the following public domain resources: https://engineering.case.edu/bearingdatacenter/download-data-file; https://github.com/ClarkGableWang/JNU-Bearing-Dataset; https://data.mendeley.com/datasets/v43hmbwxpm/1; https://www.mfpt.org/fault-data-sets/.
